# Association between stunting and fecal calprotectin levels in breastfed infants living in a rural area of the Democratic Republic of the Congo: a cross sectional study

**DOI:** 10.4314/ahs.v24i3.35

**Published:** 2024-09

**Authors:** Joe Bwija Kasengi, Ghislain Maheshe Balemba, Marius Baguma, Espoir Bwenge Malembaka, Richard Mbusa Kambale, Oreste Battisti, Paluku Bahwere, Ghislain Bisimwa Balaluka

**Affiliations:** 1 Department of Pediatrics, Hôpital Provincial Général de Référence de Bukavu (HPGRB), Bukavu, Democratic Republic of the Congo; 2 Faculty of Medicine, Université Catholique de Bukavu (UCB), Bukavu, Democratic Republic of the Congo; 3 Department of Internal Medicine, Hôpital Provincial Général de Référence de Bukavu (HPGRB), Université Catholique de Bukavu (UCB), Bukavu, Democratic Republic of the Congo; 4 Center for Tropical Diseases and Global Health (CTDGH), Université Catholique de Bukavu (UCB), Bukavu, Democratic Republic of the Congo; 5 Ecole Régionale de Santé Publique (ERSP), Faculté de Médecine, Université Catholique de Bukavu, Bukavu, Democratic Republic of Congo; 6 Department of Epidemiology, Johns Hopkins Bloomberg School of Public Health, Baltimore, USA; 7 Institute of Experimental and Clinical Research, Université Catholique de Louvain, Brussels, Belgium; 8 Département des sciences cliniques, Université de Liège, Liège, Belgium; 9 Valid International, Oxford, United Kingdom; 10 Centre de Recherche en Épidémiologie, Biostatistique et Recherche Clinique, Ecole de Santé Publique, Université Libre de Bruxelles, Brussels, Belgium

**Keywords:** Stunting, fecal calprotectin, infants, breastfeeding

## Abstract

**Background:**

Stunting is a major public health problem in low- and middle-income countries as in Democratic Republic of the Congo (DRC). Many factors, such as environmental enteric dysfunction (EED), are incriminated in the pathogenesis of stunting. EED, characterized by intestinal inflammation, can be evaluated by a non-invasive marker, the fecal calprotectin (FC). The aim of this study was to determine the concentration of FC, to compare the FC of exclusively breastfed infants with that of mixed-fed infants, and to assess the association between the stunting and the FC in infants aged 4 to 7 months.

**Methods:**

Socio-demographic, nutritional, and clinical data were collected in infants aged 4 to 7 months attending child welfare clinic activities from eight targeted rural health areas of South-Kivu province, eastern DRC. A single assay of calprotectin was performed in stool samples by Bühlmann Quantum Blue® Calprotectin Extended device.

**Results:**

A total of 240 infants (median age: 6 months, interquartile range: 5-6 months) were enrolled in this study. Among them, 41 (17.1%) were stunted. The median FC was 87 µg/g of stool. Exclusive breastfed infants had significantly higher FC compared to mixed-fed infants (median FC: 108 µg/g versus 79 µg/g, p=0.031). In addition, stunted infants had higher FC levels (107 µg/g) compared to infants with normal length for age (82 µg/g) but without reaching the statistical significance level (p= 0.41).

**Conclusion:**

This study shows higher FC levels in exclusively breastfed and in stunted infants living in rural areas of South-Kivu. Further studies are needed to assess the prevalence of EED and its association with stunting in the DRC.

## Introduction

Stunting, or being too short for one's age, is defined as having a height more than two standard deviations below the World Health Organization (WHO) child growth standards median. It is characterized by an impaired growth and development that children experience from poor nutrition, repeated infections, and inadequate psychosocial stimulation^1^. Stunting remains a major public health problem in low and middle income countries due to its short- and long-term consequences^2^. Its prevalence exceeds 30 % in some of these countries, like in the Democratc Republic of the Congo (DRC)^3^,^4^. The latest demographic health survey (DHS) reported a stunting prevalence of 42% in the DRC5. In this DHS, the province of South-Kivu, in the eastern part the DRC, had a prevalence higher than the national average, i.e. 48% 5.

The pathophysiology of stunting is not yet fully understood but available evidence points toward a multifactorial origin with genetic, maternal nutrition and early infancy feeding practices factors involved. Recently, it has been mentioned that a pathological entity called “environmental enteric dysfunction” (EED), characterized by a sub-clinical inflammation of intestinal mucosa, disrupting its digestive and absorptive functions and also plays a role in the pathogeny of stunting^6^,^7^. It was reported that the prevalence of intestinal mucosa abnormalities observed in EED was high in the South-Kivu region, especially among children suffering from acute malnutrition^8^. However, this study did not include infants under 6 months of age, as the break in the growth curve generally occurs from the 3rd to 4th month of age^9^. Theoretically, at 3-4 months of age, infants should be protected from EED by exclusive breastfeeding but early introduction of complementary foods is still common in DRC^5^,^10^.

It is now possible to detect the presence of intestinal inflammation by non-invasive techniques, particularly by measuring calprotectin in the stool. Despite the controversy over the significance of elevated concentration of calprotectin in the stools of infants and the effect of the type of infant feeding on this same level, the determination of fecal calprotectin (FC) remains among the reference tests for the evaluation of intestinal inflammation^11^,^12^.

This study aimed to determine the concentration of FC in apparently healthy infants aged 4 to 7 months, to compare the FC levels of exclusively breastfed infants with that of mixed-fed infants, and to assess the association between FC levels and the stunting among infants aged 4 to 7 months living in rural eastern DRC.

## Methods

### Study design

This cross-sectional study was conducted in Miti-Murhesa Health Zone (MMHZ) located in the Province of South-Kivu, in eastern DRC. The MMHZ is located in the territory of Kabare, which has agriculture, livestock, and fishing as its main activities. The study was carried out in 8 health areas of the MMHZ (Kavumu, Mbayo, Kashusha, Mushunguri, Lwiro, Kahungu, Buhandahanda and Miti-Mulungu), selected because of their accessibility from the city of Bukavu, the capital-city of the province and the accessibility of their health structures for the target poulation.

### Child recruitment process and data collection

The recruitment process included community sensitization and information about the study, securing the consent from the community, identification of eligible children by the local community volunteer and the referral of eligible infants to the nearest health centers used as study sites for clinical examination and stool sampling. To ensure the representativeness of the population of MMHZ, 25-35 of the eligible infants were selected per village of the targeted health areas. Sample size was not calculated but rather defined by practical limitations related to lack of financial resources. Indeed, only a total of 300 FC assays could be performed. And to be assured of the consistency of the results, 60 samples (25 % of samples) were tested in duplicate. Since the FC values obtained appeared consistent, only the values of the first measurement were retained for these 60 (25 %) samples, and only one measurement was performed for the remaining 180 (75 %) samples. Thus, the sample size of this study was limited to the 240 infants whose stool samples could be assayed.

The eligibility criteria were: age between 4 and 7 months, birth weight ≥ 2500g, being partially or exclusively breastfed, and attending the monthly child welfare clinic activities. Infants suffering from chronic pathologies (heart disease, HIV infection, congenital malformation), those born with low birth weight, those never breastfed, and twins were excluded. Never breastfed infants were excluded since they are more likely to acquire gastrointestinal infections^13^,^14^, which can increase FC levels^15^.

For each household, demographic and clinical data were collected, as well as data on hygiene (drinking water and hand washing). For each enrolled infant, the following parameters were collected: households socio-demographic characteristics (parent's education level, nutritional status of the siblings, water and sanitation parameters), child demographic data (age, sex), child nutrition data (birth weight, weight, length, Middle-Upper Arm Circumference (MUAC), type of feeding), child morbidity (symptoms and signs of diseases within two weeks before the visit).

Weight, length, MUAC and Head circumference (HC) were measured using standard procedures. Weight was measured using a SECA hanging mechanical scale (precision: 50 g; SECA GmbH & Co, model: 310, Hamburg, Germany). Length was measured using a SECA measuring board (precision: 1 cm; SECA GmbH & Co, model: 210, Hamburg, Germany). MUAC was measured using a flexible non-stretchable MUAC tape (precision 0.1cm; IndoSurgicals Private Limited, New Delhi, India).

### Laboratory procedures: Fecal calprotectin

Under the supervision of community volunteers, mother or caregiver collected the infant's morning stools using a sterile plastic jar provided by the research team the day before the study team visit. Community volunteers as well as mothers had been previously instructed on the proper methods for collecting the stools to avoid any contamination of the sample and the collection jar. Stools were collected on absorbent diapers before being collected in the sterile plastic jars to minimize stool contamination by urines. The stool samples were then collected by the laboratory technician, labelled, stored in insulated boxes, and analyzed at the health center within four hours of collection.

The BÜHLMANN Quantum Blue® fCAL Extended device (BÜHLMANN Laboratories AG, Schönenbuch, Switzerland) was used for the determination of fecal calprotectin levels following manufacturer's instructions^16^,^17^. Briefly: Using the prefilled stool extraction tube CALEX- Cap, stool dip sampling was performed 3-5 times. The mixture was then vortexed for 30 seconds and incubated for 10 minutes at room temperature. After incubation, 60 µL stool extract was aspirated and placed on Calprotectin Lateral Flow Test Cassette Quantum Blue® fCal (reading range: 30 - 300 µg/g) which was introduced into Quantum Blue® Reader and the result was read after 12 minutes. In the present study, we used a FC assay kit that was limited to ranges of 30 to 300 µg/g of stool. FC values less than 30 and greater than 300 were respectively truncated at 30 and 300 µg/g of stool.

### Operational definitions

Nutritional status was assessed using the weight-for-length (WLZ), length-for-age (LAZ), and weight-forage (WAZ) indices, with values < -2 Z-score based on the 2006 WHO curves defining respectively wasting, stunting, and underweight^18^.

### Statistical analysis

Stata version 14 (StataCorp LLC, College Station, Texas, United States of America) was used for data analysis and WHO Anthro software version 3.2.2 (WHO, Geneva, Switzerland; Available online: http://www.who.int/growthref/tools/en/) was used for the computation of the anthropometric indices. The standards quantitative descriptive statistics were used to summarize the different variables. Frequencies and percentages were used to summarize categorical variables and medians with interquartile range (IQR) were used to summarize continuous variables. We The Mann-Whitney test was used for the comparison of medians. The statistical significance level was set at p < 0.05.

## Results

### General characteristics of study population

A total of 240 infants (median age: 6 months, IQR: 5-6 months) were included in this study. Table 1 describes the characteristics of their households and their nutritional parameters. Of notice, just over half (52%) of the infants were female and 59.2% were ≥ 6 months old. Approximately three quarters of mothers (74.2 %) have attended no formal education or just completed primary school, while only 25.8 % have reached secondary education or university. Most households of studied infants (61.7 %) had access to improved water sources but only less than half of them had hygienic toilets or appropriate hand washing practices. Two hundred and sixteen infants (90 %) had reportedly illness symptoms within the two weeks prior to the survey. Fifty-five (22.9 %) infants enrolled in the present study were exclusively breastfed. Complementary feeding was introduced before three months of age in 61.1 % (113 out of 185) of infants on non-exclusive breastfeeding.

**Table 1:** Demographic, anthropometric, and nutritional characteristics of the study population (n=240)

Continuous data^1^	Median (IQR)
Age (months)	6.0 (5.0 – 6.0)
Birth Weight (g)	3200.0 (3000.0 – 3600.0)
Weight (kg)	7.1 (6.5 – 7.8)
Length (cm)	64.0 (62.5 – 65.5)
Mid-Upper Arm Circumference (mm)	132.0 (125.0 – 139.0)
Head circumference (cm)	43.0 (42.1 – 44.0)
Weight for age Z-Score	-0.35 (-1.14 – 0.41)
Weight for length Z-score	0.51 (-0.50 – 1.26)
Length for age Z-score	-0.77 (-1.70 – -0.26)

**Categorical data^2^**	n (%)

Sex	
Male	115 (47.9)
Female	125 (52.1)
Age	
4 - 5 months	98 (40.8)
≥ 6 months	142 (59.2)
Exclusive breastfeeding	
Yes	55 (22.9)
No	185 (77.1)
Age of complementary food	
≤ 3 months	113 (61.1)
> 3 months	72 (38.9)
Mother's level of education	
No formal education or primary school	178 (74.2)
Secondary school or university	62 (25.8)
Water source	
Improved^3^	148 (61.7)
Unimproved^4^	92 (38.3)
Undernutrition	
Any type^5^	47 (19.6)
Stunting alone	41 (17.1)
Underweight alone	16 (6.7)
Wasting alone	4 (1.7)

1 Data are median (IQR)

2 Data are n (%)

3 “Improved water source” refers to a built water source

4 “Unimproved water source” refers to rain water and water drawn from rivers

5 “Any type” refers to infants with either stunting, underweight, or wasting, alone or in combination

Overall, 47 infants (19.6 %) had a compromised nutritional status (either stunting, underweight, or wasting, alone or in combination), with the prevalence of stunting (17,1 %) and underweight (6,7%) being close to the threshold for considering the condition to be of public health importance.

### Comparison of fecal calprotectin levels in different conditions

The median (IQR) FC of the 240 infants included in this study was 87 (36.5 - 192) µg/g of stool. Of notice, the 193 apparently healthy infants (i.e., those with a normal nutritional status) had a median (IQR) FC level of 79 (37 - 163) µg/g of stool. Exclusively breastfed infants had a median FC significantly higher than that observed in mix-fed infants [108.0 (40 - 245) versus 79.0 (34 - 156) µg/g of stool, p=0.031]. As well, FC levels were relatively higher in infants for whom complementary feeding was introduced before 3 months and those for whom it was initiated between 4 and 6 months of age, but the difference was not statistically significant [82.0 (35.0 - 175.0) vs 77.5 (33.0 - 147.5) µg/g of stool, p= 0.637]. Stunted, underweight, and wasted infants tended to have higher median FC levels [respectively: 107.0 (32.0 - 223.0) µg/g, 103.0 (42.0 - 249.5) µg/g, and 262.0 (134.0 - 296) µg/g] compared to infants with normal LAZ [82.0 (37.0 - 165.0) µg/g], WAZ [83.5 (36.0 - 188.0) µg/g], and WLZ [85.5 (36.0 - 181.0) µg/g], but the differences did not reach the statistical significance (Table 2). As well, no statistically significant difference was found between infants with versus without a combination of different types of undernutrition: stunting + underweight [11 infants, median FC level: 95 (40.0 – 236.0) µg/g versus 86 (36.0 - 189.0) µg/g, p=0.87], underweight + wasting [3 infants, median FC level: 292.0 (36.0 - 300) µg/g vs 87.0 (37.0 - 185.0) µg/g, p=0.87], or stunting + wasting + underweight [1 infant, FC level: 30.0 µg/g vs 87 (37.0 - 193.0) µg/g, p=0.14].

**Table 2:** Levels of fecal calprotectin in different conditions

Parameters			n	Fecal calprotectin (µg/g) *	p
Sex					
	Male		115	80.0 (37.0 - 185.0)	0.709
	Female		125	94.0 (36.0 - 193.0)	
Age					
	4 - 5 months		98	80.0 (36.0 - 193.0)	0.490
	≥ 6 months		142	89.0 (38.0 - 191.0)	
Exclusive breastfeeding					
	Yes		55	108.0 (40 - 245)	0.031
	No		185	79.0 (34 - 156)	
Time of initiation of complementary food					
	≤ 3 months		113	82.0 (35.0 - 175.0)	0.637
	> 3 months		72	77.5 (33.0 - 147.5)	
Mother's level of education					
	No formal education or primary school		62	77.5 (34.0 - 145.0)	0.217
	Secondary school or university		178	94.0 (38.0 - 203.0)	
Water source					
	Improved		92	75.5 (36.0 - 162.0)	0.498
	Unimproved		148	93.5 (36.5 - 205.0)	
Undernutrition					
	Any type				
		Yes	47	108.0 (36.0 - 236.0)	0.220
		No	193	79.0 (37.0 - 163.0)	
	Stunting				0.484
		Yes	41	107.0 (32.0 - 223.0)	
		No	199	82.0 (37.0 - 165.0)	
	Underweight				
		Yes	16	103.0 (42.0 - 249.5)	0.458
		No	224	83.5 (36.0 - 188.0)	
	Wasting				
		Yes	4	262.0 (134.0 - 296)	0.109
		No	234	85.5 (36.0 - 181.0)	

* Data are median (Interquartile range)

### Impact of recent diseases symptoms on fecal calprotectin levels

No symptoms had significantly influenced the FC level, although the median of FC was relatively higher in infants who had presented diarrhea, vomiting, and abdominal colic two weeks before the visit, as compared to asymptomatic infants (Table 3).

**Table 3:** Symptoms of diseases within two weeks and their impact on the level of fecal calprotectin

Symptoms within two weeks			n (%)	Fecal calprotectin (µg/g) *	p
	Any symptoms				
		Yes	216 (90.0)	87.5 (36.5 - 188.0)	0.842
		No	24 (10.0)	74.0 (36.5 - 223.5)	
	Rhinorrhea				
		Yes	157 (65.4)	85.0 (35.0 - 175.0)	0.376
		No	83 (34.6)	94.0 (39.0 - 232.0)	
	Cough				
		Yes	140 (58.3)	88.5 (39.5 - 196.0)	0.554
		No	100 (41.7)	80.5 (36.0 - 156.0)	
	Fever				
		Yes	109 (45.4)	89.0 (36.0 - 160.0)	0.412
		No	131 (54.6)	80.0 (37.0 - 221.0)	
	Diarrhea				
		Yes	96 (40.0)	96.5 (51.5 - 197.5)	0.069
		No	144 (60.0)	77.5 (33.0 - 173.5)	
	Abdominal pain diversification				
		Yes	86 (35.8)	98.0 (34.0 - 175.0)	0.797
		No	154 (61.2)	79.5 (37.0 - 207.0)	
	Rashes				
		Yes	89 (37.1)	97.0 (41.0 - 175.0)	0.806
		No	151 (62.9)	80.0 (36.0 - 207.0)	
	Vomiting				
		Yes	49 (20.4)	80.0 (30.0 - 185.0)	0.331
		No	191 (79.6)	87.0 (39.0 - 196.0)	

## Discussion

In the present study, the median FC was 87 µg/g of stool in 4-7-month-old infants. Compared to FC levels found in other studies in infants under 7 months, the median FC value found in our study is lower than 278 µg/g reported in urban areas in Uganda^19^, and then 217.9 µg/g and 420 µg/g found respectively in urban^20^, and rural^21^ areas in China. However, it was close to 79 µg/g of stool described in Norway^22^.

Exclusively breastfed infants had significantly higher FC compared to those with a mixed feeding. This was consistent with the findings of the majority of studies conducted elsewhere^23^–^28^. The higher FC reported in young exclusively breastfed infants (as compared to mixed-fed infants) is thought to play a protective role against various attacks on the immature intestinal mucosa up to maturation and regulation with age of epithelial tight junction proteins and other factors of intestinal immunity^20^,^23^,^30^. However, the fact that FC, used as a marker of intestinal inflammation, is higher in breastfed infants creates a paradoxical phenomenon in the literature, and several controversies still persist about this phenomenon. Some studies found no difference between FC and type of feeding^31^. Early artificial feeding of infants is thought to lead to increased transepithelial migration oneutrophils, cells in which calprotectin represents around 60% of the cytoplasmic proteins^32^.

In this study, we found a low rate of stunted infants compared to the high rate described in our region. The rate of stunting is high in under five years' children and increases with age in our study area5. We believe that the low rate of stunting observed in the population included in this study may largely be explained by that we included only infant who were still breastfed. In fact, previous study has demonstrated that exclusive breastfeeding protects young children from stunting in low-income populations^33^. FC was elevated in stunted infants in our study population. Indeed, several markers that can be measured in the stool, in particular calprotectin, myeloperoxidase, and neopterin, are used as non-invasive markers of intestinal inflammation in children in various studies^21^,^34^–^38^. These studies described intestinal inflammation in EED which is at the root of stunting in children.

Despite the association found between high FC levels and stunting in children^21^, there is no consensus in the literature whether this increased FC in infants is associated with a deleterious effect on height and/or weight growth or it rather has a protective role on the intestinal mucosa regarding various attacks, particularly microbial in young infants^11^,^39^–^41^. The pathogenesis of stunting remains multifactorial and EED would be one of these factors^39^,^42^,^43^. Indeed, EED is a chronic inflammation of the small intestine characterized by villous atrophy, crypt hyperplasia and increased intestinal permeability^44^. Chronic intestinal inflammation is believed to be linked to a vicious cycle between undernutrition, infection, and poor environmental conditions.

In young infants living in low- and middle-income countries, this vicious cycle is thought to be due to continued exposure to fecal-oral contamination by microbes, the repeated intestinal infections they cause, and undernutrition^45^. Indeed, in EED, it has been described an increased bacterial proliferation in the small intestine leading to local inflammation and to an alteration of the intestinal barrier, digestion and nutrient absorption functions^46^–^49^. In addition, intestinal inflammation in children defined by high fecal calprotectin has been associated with a low level of Insulin Growth Factor-1 in infants under 18 months in Zimbabwe^50^, with a low height for age index age in Kenya and Bangladesh^51^,^52^.

Some limitations should be considered when interpreting the findings of this study: Due to logistical and financial constraints, the study sample size was limited to 240 infants as only 240 stool samples could be assayed for FC. Thus, the relatively small sample size, especially the small number of stunted infants (only 41 infants were stunted), may have influenced the findings of this study. In addition, FC measurements were done in duplicate for only 25 % of stool samples, while only one FC measurement was done for the remaining 75 % of samples. As well, inflammatory blood tests or parasitic/microbiological investigations were not performed on collected stool samples in order to rule out potential still ongoing infections in infants whose mothers or caregivers reported some symptoms within two weeks before the medical visit. Finally, the fact that symptoms were reported by infants' mothers of caregivers may have been source of misinterpretation of some symptoms. For example, 4 to 6-month-old infants are prone to physiological gastro-esophageal reflux which can be difficult to distinguish from vomiting, especially for parents/caregivers with no medical training. Despite these limitations, to our knowledge, this study is the first to be conducted in the DRC in infants under seven months of age to determine the concentration of FC, to compare the FC levels in breastfed and mixed-fed infants, and to assess the association between FC of stunting in infants of in this age-group. A study with sequential dosing of FC throughout the height-weight growth and the gradual dietary diversification of infants may help to better understand the influence of breastfeeding and of stunting on infants' FC levels.

## Conclusion

This study shows higher FC levels in exclusively breastfed and in stunted infants living in rural areas of South Kivu, in the East of the DRC. Further studies are needed to better understand the influence of breastfeeding and of stunting on infants' FC levels and to assess the prevalence of EED in our region.

## Data Availability

The data presented in this study are available on request from the corresponding author.

## Abbreviations

DRC: Democratic Republic of the Congo
DHS: Demographic Health Survey
EED: Environmental Enteric Dysfunction
FC: Fecal Calprotectin
HIV: Human Immunodeficiency Virus
HC: Head circumference
IQR: Interquartile range
LAZ: Length-for-age Z-score
MMHZ: Miti-Murhesa Health Zone
MUAC: Middle-Upper Arm Circumference
WAZ: Weight-for-age Z-score
WLZ: Weight -for-Height Z-score

## References

[1] WHO (2015). Stunting is a nutshell [Internet].

[2] OMS (2012). Le retard de croissance.

[3] Unicef/WHO/The World Bank (2019). Levels and Trends in Child malnutrition - Unicef WHO The World Bank Joint Child Malnutrition Estimates, key findings pf the 2019 edition. Unicef.

[4] WHO african region (2017). Nutrition in the WHO african region.

[5] Institut national de la statistique du ministère du Plan de la RDC (2019). Enquête par grappes à indicateurs multiples, 2017-2018. Rapport de résultats de l'enquête.

[6] Lauer JM, McDonald CM, Kisenge R, Aboud S, Fawzi WW, Liu E (2019). Markers of Systemic Inflammation and Environmental Enteric Dysfunction Are Not Reduced by Zinc or Multivitamins in Tanzanian Infants: A Randomized, Placebo-Controlled Trial. J Pediatr.

[7] McKay Sue, Gaudier Estelle, Campbell David I, Prentice Andrew M, RA (2010). Environmental enteropathy: new targets for nutritional interventions. Int Health.

[8] Brasseur D, Goyens P, Vis HL (1992). [Enzymes and histology of the intestinal mucosa of breast-fed African infants]. Ann Pediatr (Paris).

[9] Tonglet R, Katulanya-Isu, Chiabrera F, Dramaix M, Hennart P (1991). Pattern of attained growth in 0 to 5 year-old children from Kivu (Zaire). Ecol Food Nutr.

[10] Taylor SN, Basile LA, Ebeling M, Wagner CL (2009). Intestinal Permeability in Preterm Infants by Feeding Type: Mother's Milk Versus Formula. Breastfeed Med.

[11] Lisowska B (2015). Changes in Physiology and Pathophysiology of Calprotectin Excretion from Neonate to Adult. J Mol Biomark Diagn.

[12] Savino F, Castagno E, Calabrese R, Viola S, Oggero R, Miniero R (2010). High faecal calprotectin levels in healthy, exclusively breast-fed infants. Neonatology.

[13] Crane RJ, Parker EPK, Fleming S, Gwela A, Gumbi W, Ngoi JM (2022). Cessation of exclusive breastfeeding and seasonality, but not small intestinal bacterial overgrowth, are associated with environmental enteric dysfunction: A birth cohort study amongst infants in rural Kenya. eClinicalMedicine.

[14] Quigley MA, Kelly YJ, Sacker A (2007). Breastfeeding and hospitalization for diarrheal and respiratory infection in the United Kingdom Millennium Cohort Study. Pediatrics.

[15] Gisbert JP, McNicholl AG (2009). Questions and answers on the role of faecal calprotectin as a biological marker in inflammatory bowel disease. Dig liver Dis.

[16] BÜHLMANN Laboratories (2022). CALEX® Cap. For In Vitro Diagnostic Use [Internet].

[17] BÜHLMANN Laboratories (2021). Quantum Blue® fCAL extended: Quantitative Lateral Flow Assay [Internet].

[18] World Heath Oganisation: Multicentre Growth Reference Study Group (2006). WHO Child Growth Standards: Length/Height for-Age, Weight-for-Age, Weight-for-Length, Weight-for-Height and Body Mass Index-for-Age: Methods and Development.

[19] Hestvik E, Tumwine JK, Tylleskar T, Grahnquist L, Ndeezi G, Kaddu-Mulindwa DH (2011). Faecal calprotectin concentrations in apparently healthy children aged 0-12 years in urban Kampala, Uganda: A community-based survey. BMC Pediatr.

[20] Li F, Ma J, Geng S, Wang J, Liu J, Zhang J (2015). Fecal Calprotectin Concentrations in Healthy Children Aged 1-18 Months. PLoS One.

[21] Liu JR, Sheng XY, Hu YQ, Yu XG, Westcott JE, Miller L V (2012). Fecal calprotectin levels are higher in rural than in urban Chinese infants and negatively associated with growth. BMC Pediatr.

[22] Rugtveit J, Fagerhol MK (2002). Age-dependent variations in fecal calprotectin concentrations in children [2]. Journal of Pediatric Gastroenterology and Nutrition.

[23] Dorosko SM, MacKenzie T, Connor RI (2008). Fecal Calprotectin Concentrations Are Higher in Exclusively Breastfed Infants Compared to Those Who Are Mixed-Fed. Breastfeed Med.

[24] Li F, Ma J, Geng S, Wang J, Ren F, Sheng X (2014). Comparison of the different kinds of feeding on the level of fecal calprotectin. Early Hum Dev.

[25] Moussa R, Khashana A, Kamel N, Elsharqawy SE (2016). Fecal calprotectin levels in preterm infants with and without feeding intolerance. J Pediatr (Rio J).

[26] Asgarshirazi M, Shariat M, Nayeri F, Dalili H, Abdollahi A (2017). Comparison of fecal calprotectin in exclusively breastfed and formula or mixed fed infants in the first six months of life. Acta Med Iran.

[27] Velasco Rodríguez-Belvís M, Palomino Pérez LM, García Salido A, Plata Fernández C, Muñoz Codoceo RA (2020). Letter to the editor: The role of breast milk in fecal calprotectin levels in healthy infants. Early Hum Dev.

[28] Günaydın Şahin BS, Keskindemirci G, Özden TA, Durmaz Ö, Gökçay G (2020). Faecal calprotectin levels during the first year of life in healthy children. J Paediatr Child Health.

[29] Campeotto F, Baldassarre M, Butel MJ, Viallon V, Nganzali F, Soulaines P (2009). Fecal calprotectin: Cutoff values for identifying intestinal distress in preterm infants. J Pediatr Gastroenterol Nutr.

[30] Groer M, Ashmeade T, Louis-Jacques A, Beckstead J, Ji M (2016). Relationships of Feeding and Mother's Own Milk with Fecal Calprotectin Levels in Preterm Infants. Breastfeed Med.

[31] Rodríguez-belvís MV, Francisco J, Bris V, Plata C, Pd F, García-salido A (2020). Normal fecal calprotectin levels in healthy children are higher than in adults and decrease with age.

[32] Berstad A, Arslan G, Folvik G (2000). Relationship between Intestinal Permeability and Calprotectin Concentration in Gut Lavage Fluid. Scand J Gastroenterol.

[33] Hadi H, Fatimatasari F, Irwanti W, Kusuma C, Alfiana RD, Ischaq Nabil Asshiddiqi M (2021). Exclusive breastfeeding protects young children from stunting in a low-income population: A study from Eastern indonesia. Nutrients.

[34] Dhaliwal J, Leach S, Katz T, Nahidi L, Pang T, Lee JM (2015). Intestinal inflammation and impact on growth in children with cystic fibrosis. J Pediatr Gastroenterol Nutr.

[35] Lunn PG, Northrop-Clewes CA, Downes RM (1991). Intestinal permeability, mucosal injury, and growth faltering in Gambian infants. Lancet.

[36] Campbell DI, McPhail G, Lunn PG, Elia M, Jeffries DJ (2004). Intestinal inflammation measured by fecal neopterin in Gambian children with enteropathy: association with growth failure, Giardia lamblia, and intestinal permeability. J Pediatr Gastroenterol Nutr.

[37] Petri WA, Naylor C, Haque R (2014). Environmental enteropathy and malnutrition: Do we know enough to intervene?. BMC Med.

[38] Canani RB, Rapacciuolo L, Romano MT, De Horatio LT, Terrin G, Manguso F (2004). Diagnostic value of faecal calprotectin in paediatric gastroenterology clinical practice. Dig Liver Dis.

[39] Campbell RK, Schulze KJ, Shaikh S, Raqib R, Wu LSF, Ali H (2018). Environmental enteric dysfunction and systemic inflammation predict reduced weight but not length gain in rural Bangladeshi children. Br J Nutr.

[40] Harper KM, Mutasa M, Prendergast AJ, Humphrey J, Manges AR (2018). Environmental enteric dysfunction pathways and child stunting: A systematic review. PLoS Negl Trop Dis.

[41] Liu JZ, Jellbauer S, Poe AJ, Ton V, Pesciaroli M, Kehl-Fie TE (2012). Zinc sequestration by the neutrophil protein calprotectin enhances salmonella growth in the inflamed gut. Cell Host Microbe.

[42] Syed S, Yeruva S, Herrmann J, Sailer A, Sadiq K, Iqbal N (2018). Environmental enteropathy in undernourished Pakistani children: Clinical and histomorphometric analyses. Am J Trop Med Hyg.

[43] George CM, Burrowes V, Perin J, Oldja L, Biswas S, Sack D (2018). Enteric Infections in Young Children are Associated with Environmental Enteropathy and Impaired Growth. Trop Med Int Heal.

[44] Watanabe K, Petri WA (2016). Environmental Enteropathy: Elusive but Significant Subclinical Abnormalities in Developing Countries. EBioMedicine.

[45] Kosek MN, Ahmed T, Bhutta Z, Caulfield L, Guerrant R, Houpt E (2017). Causal Pathways from Enteropathogens to Environmental Enteropathy: Findings from the MAL-ED Birth Cohort Study. EBioMedicine.

[46] Donowitz JR, Petri WA (2015). Pediatric small intestine bacterial overgrowth in low-income countries. Trends in Molecular Medicine.

[47] Donowitz JR, Haque R, Kirkpatrick BD, Alam M, Lu M, Kabir M (2016). Small intestine bacterial overgrowth and environmental enteropathy in Bangladeshi children. MBio.

[48] Kukuruzovic RH, Brewster DR (2002). Small bowel intestinal permeability in Australian aboriginal children. J Pediatr Gastroenterol Nutr.

[49] Vonaesch P, Morien E, Andrianonimiadana L, Sanke H, Mbecko J (2018). Stunted childhood growth is associated with decompartmentalization of the gastrointestinal tract and overgrowth of oropharyngeal taxa.

[50] Prendergast AJ, Rukobo S, Chasekwa B, Mutasa K, Ntozini R, Mbuya MNN (2014). Stunting is characterized by chronic inflammation in zimbabwean infants. PLoS One.

[51] Jones KDJ, Hünten-Kirsch B, Laving AMR, Munyi CW, Ngari M, Mikusa J (2014). Mesalazine in the initial management of severely acutely malnourished children with environmental enteric dysfunction: A pilot randomized controlled trial. BMC Med.

[52] Naylor C, Lu M, Haque R, Mondal D, Buonomo E, Nayak U (2015). Environmental Enteropathy, Oral Vaccine Failure and Growth Faltering in Infants in Bangladesh. EBioMedicine.

